# Investigating time-efficiency of forward masking paradigms for estimating basilar membrane input-output characteristics

**DOI:** 10.1371/journal.pone.0174776

**Published:** 2017-03-29

**Authors:** Michal Fereczkowski, Morten L. Jepsen, Torsten Dau, Ewen N. MacDonald

**Affiliations:** 1 Elektro, DTU, Kgs. Lyngby, Denmark; 2 Widex A/S, Copenhagen, Denmark; Universidad de Salamanca, SPAIN

## Abstract

It is well known that pure-tone audiometry does not sufficiently describe individual hearing loss (HL) and that additional measures beyond pure-tone sensitivity might improve the diagnostics of hearing deficits. Specifically, forward masking experiments to estimate basilar-membrane (BM) input-output (I/O) function have been proposed. However, such measures are very time consuming. The present study investigated possible modifications of the temporal masking curve (TMC) paradigm to improve time and measurement efficiency. In experiment 1, estimates of knee point (KP) and compression ratio (CR) of individual BM I/Os were derived without considering the corresponding individual “off-frequency” TMC. While accurate estimation of KPs was possible, it is difficult to ensure that the tested dynamic range is sufficient. Therefore, in experiment 2, a TMC-based paradigm, referred to as the “gap method”, was tested. In contrast to the standard TMC paradigm, the maker level was kept fixed and the “gap threshold” was obtained, such that the masker just masks a low-level (12 dB sensation level) signal. It is argued that this modification allows for better control of the tested stimulus level range, which appears to be the main drawback of the conventional TMC method. The results from the present study were consistent with the literature when estimating KP levels, but showed some limitations regarding the estimation of the CR values. Perspectives and limitations of both approaches are discussed.

## Introduction

The human auditory system is complex and nonlinear and a potential hearing loss can result from deficits at various auditory processing stages, even in the case of the most typical, sensorineural hearing loss. Currently, the main method used to categorize hearing loss is pure tone audiometry and the results are presented in terms of the audiogram reflecting pure-tone sensitivity [1]. While useful, it is not sufficient on its own to fully predict supra-threshold perception and performance of individual hearing-impaired (HI) listeners (e.g., [2]). In other words, two individuals with similar audiograms can differ widely on perceptual and hearing aid outcome measures (e.g., [3]). Thus, the development of improved individualized diagnostic measures are needed to allow researchers to better characterize HI test subjects. With these measures, it will be possible for researchers to increase the homogeneity of test groups and/or allow more fine-grained investigations into the perceptual consequences of hearing loss from different etiologies.

Intact functioning of the basilar membrane (BM) in the cochlea is crucial for proper auditory perception. In a normal cochlea, outer hair cells (OHCs) sharpen the tuning and amplify the mechanical response of the BM. When OHCs are damaged, the amplification is reduced, leading to a decreased sensitivity (i.e., higher thresholds), but also decreased frequency selectivity and reduced BM compression [4]. Thus, OHC loss goes beyond just elevated thresholds. While the total hearing loss can be measured by the audiogram, it has been challenging to develop rapid tests that estimate the portion of hearing loss related to reduced or absent OHC function and the related alterations of the BM characteristics.

**Fig 1:**
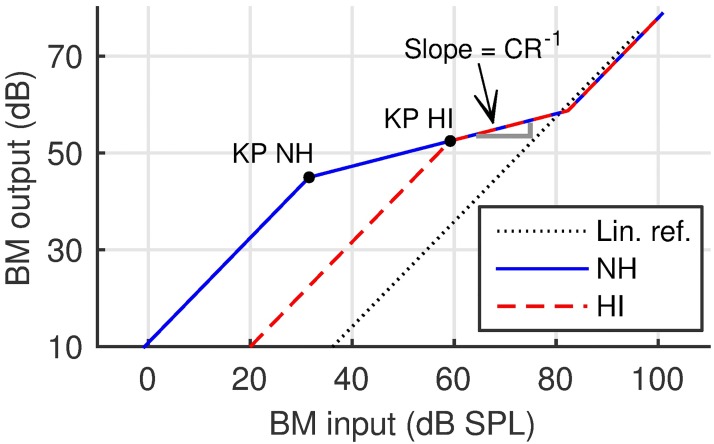
Sketch of basilar membrane I/O responses. Sketch of the basilar membrane (BM) input/output (I/O) responses, at a single frequency, for a normal-hearing (NH—solid blue line) and a hypothetical hearing-impaired (HI) listener with mild-to-moderate hearing loss (dashed red line). The curves coincide for input levels greater than 60 dB SPL, hence the maximum compression ratio is same in both cases. The dotted line represents the linear reference.

Consider the response of a single point along the BM. Fig 1 shows a sketch of I/O functions of such a point tuned to a medium frequency (e.g., 2–4 kHz). The abscissa shows the sound pressure level of a stimulating pure tone, with a frequency that matches the characteristic frequency of the operating point of the BM. Since the outer and middle ear exhibit approximately linear transfer functions, the input to the BM is a linear transformation of the input to the outer ear. The ordinate shows the response of the BM to the stimulation. The solid blue curve in Fig 1 presents the idealized BM I/O function in the normal-hearing (NH) system. The dashed red curve represents the idealized BM I/O function for a hypothetical case of a mild-to-moderate HL, as proposed by [5]. In this case, the maximum compression has been shown to be equal to that in NH listeners and both BM I/Os coincide at input levels above 60 dB SPL. The difference between the lower compression threshold (i.e., the knee-point, KP) level for a HI listener and the average KP level for NH listeners was argued to be directly related to the reduction of the maximum cochlear gain at the tested frequency [5]. While KP has been shown to be strongly correlated to the hearing threshold at the corresponding frequency [5, 6], the maximum compression ratio has been shown to be both uncorrelated with the hearing threshold [5, 7] and a significant predictor of speech intelligibility in speech-shaped noise. [8]. Therefore, this study focuses on estimating compression in individual listeners.

**Fig 2:**
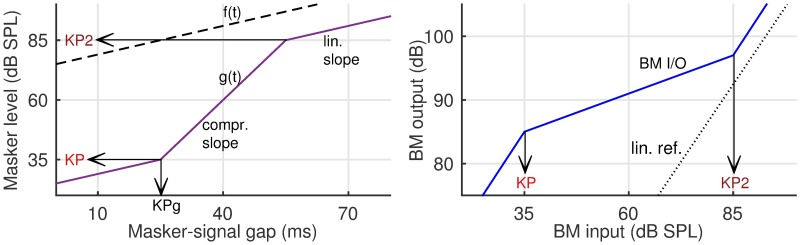
Derivation of basilar membrane I/O responses from temporal masking curves. Left panel: Sketch of temporal masking curves (TMCs). The first and third sections of the on-frequency TMC, g(t), correspond to the linear region of BM I/O while the second section corresponds to the compressive region. The knee point (KP) level, i.e. the masker level at which the slope of g(t) changes from lower to higher is indicated by an arrow. When the masker level exceeds the KP level, the increase in masker level with masker-signal gap has to overcome the BM compression, hence the steeper slope. The slopes of the first and third sections of the on-frequency TMC are set similar to the slope of the off-frequency curve, f(t). Note, that it is possible to estimate the KP level based on the on-frequency curve. Two additional arrows indicate the KPg—the gap corresponding to KP and KP2 –the masker level above which BM response returns to linearity. Right panel: Sketch of the BM I/O function (solid blue line) derived from the TMCs shown in the left panel. Masker levels of g(t) and f(t) define the ordinate and abscissa, respectively. The arrows point towards the KP and KP2 levels. The mid-section of the curve describes the compressive response, while the two outer sections correspond to the linear response of the BM. The dotted curve reflects the linear reference.

Several psychophysical methods have been developed to measure BM I/O characteristics. The authors of [9] proposed a forward masking paradigm based on measurements of growth of masking (GOM). This paradigm differed from previous approaches using simultaneous masking (e.g., [10]). An alternative paradigm, using temporal masking curves (TMC), was proposed by [11] where the signal was fixed at a low level (10 dB sensation level, SL) and the masker-signal interval was varied. Two conditions were distinguished: in the “on-frequency” condition, the masker and the signal are pure tones of same frequency whereas in the “off-frequency” condition, the frequency of the masker is set well below the signal’s frequency (e.g., 60%) and the masker is assumed to be processed linearly at the site along the BM corresponding to the signal. Based on the TMC paradigm, the BM I/O is then derived from the off-frequency and on-frequency thresholds paired according to the respective masker-signal interval. Fig 2 presents a sketch of the derivation procedure.

Masker level values of the off-frequency TMC define the output axis of the BM I/O curve, while values of the on-frequency curve define the input axis. The rationale is that, for each particular masker-signal interval, both maskers are equally effective, as they both mask the 10 dB SL signal. Assuming that the off-frequency masker is processed linearly at the site of the BM corresponding to the signal, it is used as a linear reference for the compressively processed on-frequency masker. Hence, the I/O curve only shows the change of BM activity with change of the input and is not scaled in absolute units.

In its standard form, the TMC method is very time consuming Extensive training is often required before listeners reach stable performance levels. Further, many measurement points are necessary to reliably estimate a BM I/O curve [12]. As a result, the standard TMC is not feasible for use as a screening or diagnostic tool to better characterize HI participants in listening experiments.

The purpose of the present study was to evaluate modifications of the TMC paradigm in order to, make it feasible for use as a diagnostic or screening tool in research. TMCs were measured in NH and HI listeners, using a single-interval up-down (SIUD) method proposed by [13]. Experiment 1 explored whether measuring the on-frequency TMC alone could be sufficient to estimate the KP level and the maximum CR of the BM I/O curve. In Experiment 2, a modification of the TMC paradigm is proposed, inspired by [14]. By using the masker level as a parameter instead of the masker-signal interval, the method allows for measuring compression of the BM I/O characteristic in a pre-selected level range, e.g. between 30–40 and 90 dB SPL, which is the region of compressive operation in NH listeners [2]. Other parts of the full I/O curve would not be required to be “sampled”. Thus, the modifications focus on measuring only the points necessary for estimating the maximum compression of the BM I/O.

## Experiment 1: I/O functions derived from standard TMCs

### Method

#### Listeners

Individual ears were tested from 10 NH and 10 HI listeners. The NH group consisted of 4 females and 6 males with a mean age of 26.6 years and all had thresholds of less than 20 dB HL at all frequencies (250–8000 Hz). The HI group consisted of 5 females and 5 males with a mean age of 58.7 years, all with a mild-to-moderate (25–70 dB HL) sensorineural hearing loss, based on their audiogram. The first author participated in the HI group (Listener 19). None of the HI listeners showed an air-bone conduction gap larger than 10 dB at any tested frequency. All listeners provided written informed-consent and the study was approved by the National Research Ethics Committee of Denmark.

#### Stimuli and procedure

The maskers used in the experiment were 200 ms pure tone stimuli gated with 5 ms raised cosine ramps. The signals were Hanning windowed, 16 ms pure tones with no steady-state portion. As the signal levels in the TMC paradigm are specified in terms of SL, absolute thresholds for the signals were measured first. Subsequently, on- and off-frequency TMCs were collected. Masker-signal intervals (i.e., gaps) were chosen from the set of 2, 5, 10, 15, 20, 30, 40, 50, 60 and 70 ms. The signal level was set to 12 dB SL, in order to assure salience and limit possible confusion with the on-frequency masker [7]. Three repetitions were run. If a measured threshold exceeded 85 dB SPL (95 for HI subjects) for any of the gaps, this and larger intervals were no longer used for the listener and frequency, to avoid the problem of stimuli with uncomfortable loudness. Three frequencies were tested, f = 1, 2 and 4 kHz. The off-frequency condition was taken as 60% of the signal frequency and was tested only for the 4 kHz signal. All stimuli were generated on a PC running Matlab with a 24 bit RME soundcard. Presentation was monaural via Sennheiser HD580 headphones in a double-walled, sound-attenuating booth.

**Fig 3:**
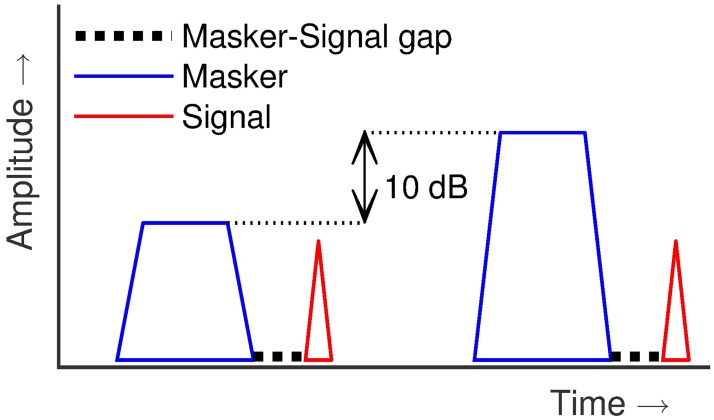
Sketch of the time course of a stimulus. Sketch of a time course of a stimulus presented in a single trial. The only difference between the two masker-signal pairs is that the first masker level is 10 dB lower than second masker level. The first masker-signal pair serves as a reference cue to the listener and the listener’s response is based on the second pair.

The SIUD threshold tracking method, used here, is based on that of [13]. Briefly, it is a 1-up-1-down, yes/no procedure. Hence, it estimates the 50% threshold on a psychometric function. Fig 3 presents a sketch of the stimulus presented in a single trial. While it is denoted as single interval, two masker-signal pairs are presented in each trial. Listeners were instructed to use the first pair as a reference cue and base their judgement on the second pair. Within a single run, the masker-signal interval, the gap, was fixed at a single value (e.g., 10 ms) and the masker level was varied. The initial step size of 10 dB was reduced to 6 dB after 2 reversals, and further reduced to 2 dB after the next 2 reversals. Up to 12 responses were recorded using the smallest step size and the mean of the corresponding masker levels was taken as the threshold estimate. “Catch trials” [15] where the signal tone was absent, were presented at random, to prevent listeners from adopting a strategy of always reporting the signal presence. If a listener reported hearing a signal tone during a catch trial, the run was restarted and the listener was instructed to only respond “yes” when he/she was sure about the presence of the signal.

#### Training

Listeners were trained using a same-different paradigm, which is often used in experiments where the cues in the stimuli are difficult to describe [16]. The “same” stimulus consisted of two identical masker-signal pairs, presented in succession. The masker-signal interval was set to 15 ms, the masker level was set 10 dB above the audiometric threshold at corresponding frequency and the signal tone was fixed at 12 dB SL. The “different” stimulus was identical, except that the second signal tone was absent. If the listener could perceive the difference between the “same” and “different” stimuli, the masker level was increased, otherwise decreased.

As soon as the listener could clearly perceive the difference, he/she was asked to focus on the second masker-signal pair and to judge whether the second signal was present or absent. The masking level was fixed and examples with and without the signal were presented randomly. After six consecutive correct responses (corresponding to a chance level of 1.6%), the listener was tested to determine if he/she was adequately trained. Three runs using an SIUD method for a single, 10 ms, masker-signal gap were conducted. If the standard deviation of the three threshold estimates was less than 3 dB, training was finished. Otherwise, additional instructions were provided and specific problems/questions of the participant were addressed. Further training and testing were repeated until the 3 dB criterion was met.

#### Data analysis

For each tested frequency for each listener, an on-frequency TMC was approximated by a three-section fit (i.e., three straight lines that intersected at knee points; see the left panel of Fig 2 for a schematic of the fit). This fitting model directly corresponds to three-section fits to BM I/Os (e.g., [5]) where the sections correspond to the linear-compressed-linear sections typical of BM I/O functions [7]. The mean and standard error of the fitted parameters were estimated using the bootstrapping method of [17]. In the variant used here, a single fit to the complete set of 30 TMC threshold estimates (3 estimates per gap x 10 gaps) was provided and the fitted value of the parameter under investigation was used as the estimate of the mean. Subsequently, fits were performed to all 30 possible 29-element sets in order to estimate the standard deviation of the mean.

Each three-section fit was defined by five parameters: a linear-processing slope, a compressive-processing slope, KPg, KP, and KP2. The linear-processing slope corresponded to the slope of the 1^st^ and the 3^rd^ sections. For these portions of the TMC, it was assumed that the masker is processed linearly. The compressive-processing slope corresponds to the slope of the 2^nd^ section. The two parameters KPg and KP are the masker-signal gap and masker level, respectively, of the point where the 1^st^ and 2^nd^ sections intersect. KP2 corresponds to the masker level where the 2^nd^ and 3^rd^ sections intersect.

A constrained minimization routine of the Akaike Information Criterion (AIC; e.g., [18]) of the fit to the collected data-points was run. The AIC is used in optimization problems to avoid model over-fitting, since it increases with increasing fit error and with increasing number of model parameters. The constraints were chosen based on the following assumptions regarding human BM I/O functions. The linear-processing slope of a TMC was assumed to be positive and smaller than the corresponding compressive-processing slope. The compression ratio estimates were bounded between 1.05 and 10. Given the noise in the collected data and standard deviations of the calculated fittings, it was not possible to estimate the compression ratio with a precision higher than one decimal place. The maximum values for CR reported in the literature vary from 4–6 in NH listeners and are not larger in HI listeners. Thus, the upper limit of 10 was chosen to assure some head room for the estimates. As the BM I/O and TMCs are assumed to be monotonic, KP2 was assumed to be greater than KP. It was reported in [3] that, in some cases, there is a return to linear behaviour of BM I/O functions above 85 dB SPL. Based on this, the KP level was allowed to vary between 0 and 100 dB SPL, while KP2 was always greater than KP and varied between 75 and 120 dB SPL. In some cases, the data did not support one or two of the sections (i.e., they could be better modelled as two sections or a single line, respectively). These cases were identified by comparing the fitted knee point gap-values to the span of masker-signal gaps tested. If a fitted KP or KP2 lay outside that span, it was discarded. In such cases, the number of parameters needed to fit a given TMC was reduced and the AIC adjusted. Thus the fitting procedure was flexible (in that it could fit among one-, two- and three-section functions) and at the same time the risk of over-fitting was decreased. Two special cases occurred when both fitted knee points lay above or below the data range. In the first case, it was assumed that only the low-level linear portion of the BM I/O was estimated and the fitted CR value was discarded. In the second case, it was assumed that the high-level linear portion of the BM I/O was estimated and the fitted CR value was set to 1. While three section fits were used for TMCs by [5] and [6], they used fewer constraints.

Within each single on- and off-frequency TMC collected for a listener, a distribution of distances between threshold estimates collected for each single gap (raw data) and the corresponding mean threshold estimates was observed. The standard deviation of such distribution, σ, was then used as an outlier criterion. Specifically, a data point was considered an outlier if its individual distance to the corresponding mean exceeded 3σ.

Off-frequency slopes were fitted using linear regression. The regression parameters were used to extrapolate the off-frequency data so that it matched the masker-signal interval range of the on-frequency data. In this way, larger portions of the BM I/O functions could be estimated. BM I/Os were obtained by pairing points from the on- and off-frequency TMCs, as shown in Fig 2. With the exception of the linear-processing slope, the BM I/O curves were fitted using the same five parameters and constraints used in fitting the TMCs. For BM I/O fits, the linear-processing slope was fixed at 1 dB/dB.

### Results

**Fig 4:**
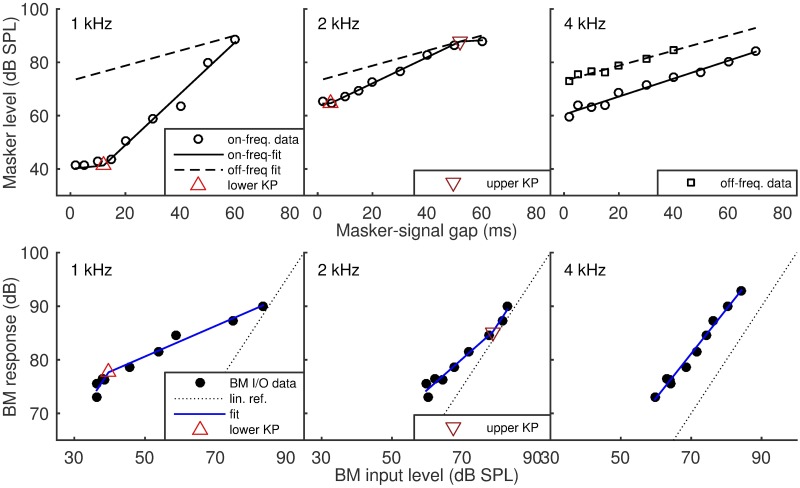
Comparison of the measured and fitted TMCs and corresponding BM I/Os in a single listener. Top three panels: comparison of the measured and fitted TMCs in a single listener at three signal frequencies– 1, 2 and 4 kHz. (left, middle and right panel, respectively). Open symbols (circles and squares) show mean on- and off-frequency masker-thresholds, while solid and dashed-lines show the corresponding fits. Triangles and inverted triangles show the fitted knee points. Bottom three panels: corresponding BM I/O data and fits. Note that while the fitted knee-point in the lower-left panel coincides with that fitted in the upper-left panel, this is not the case in the middle panels. This is the consequence of fitting the TMCs and BM I/Os independently.

The top three panels of Fig 4 present TMC data collected for one of the HI listeners (L20), together with the corresponding three-section fits. The data for this listener was chosen because the maximum slope of on-frequency TMC varies substantially across frequencies—from much steeper (left panel) to essentially same as the slope of the off-frequency curve (right panel). Moreover, different panels show how one-, two- and three-section fits may be most appropriate in different cases. Overall the coincidence of the fits with the data suggests that the fitting procedure provides flexibility to accommodate different scenarios.

The bottom three panels show the corresponding BM I/O data and fits. Note that while input level ranges of individual BM I/Os match the masker-level ranges of the on-frequency TMCs, the BM I/O output level ranges exceed those of the off-frequency maskers. This is a consequence of extrapolating the off-frequency curves, before fitting BM I/O curves. As in the previous case, the fits appear to be a good approximation of the data given that the maximum number of free parameters was 4.

The first hypothesis tested was that, for an individual listener, the slope of the first and third sections fitted to the on-frequency TMC data could be used as an estimate of the off-frequency slope. For each individual, the slopes from the sections of the on-frequency TMCs assumed to involve linear processing were averaged across the three frequencies tested and compared with the slope of the off-frequency TMC. This approach relies on two assumptions. The first is that the decay of forward masking is frequency-independent [11, 19] and thus, the low-level segments of TMCs that reflect linear processing should show slopes similar to that of the off-frequency reference. The second assumption is that the off-frequency slope itself reflects linear processing at BM. This might not be the case when the frequency of the off-frequency masker is more than 1.6 kHz for a 4-kHz signal [20]. However, measuring off-frequency maskers separated by more than an octave leads to very high masker-levels and limits the dynamic range of the obtained curve. [21] suggested that even a 1.5 kHz masker could be subject to compression at the 4 kHz place of the BM, at least in NH listeners. However, this off-frequency compression could be retro-cochlear in origin, which would not affect the shape of BM I/O.

**Fig 5:**
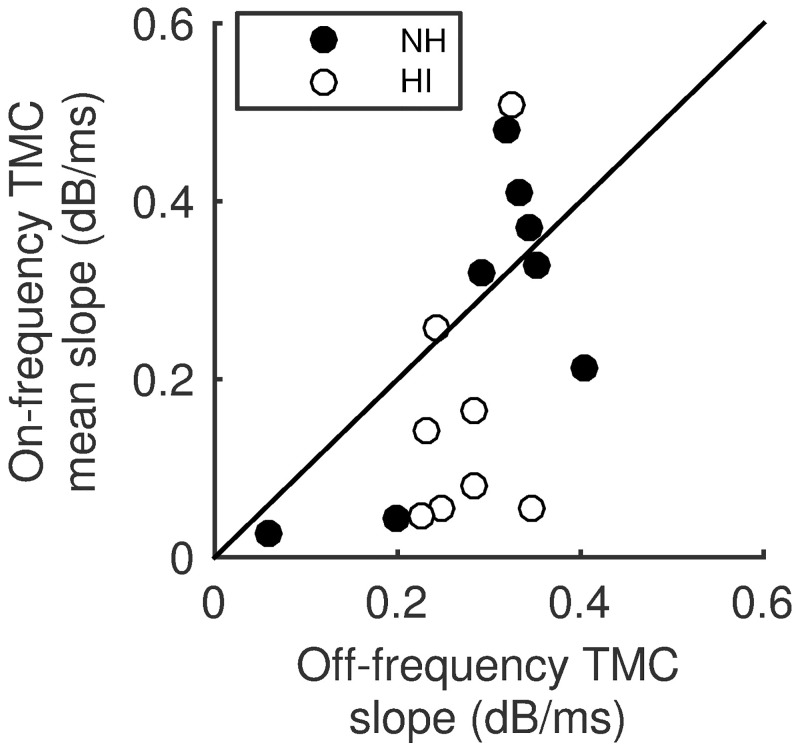
Comparison of the estimated slopes of temporal masking curves. Comparison of TMC slopes corresponding to linear processing on BM I/O. The off-frequency slope and the mean of corresponding on-frequency slopes are approximately equal, on average, and significantly correlated.

A scatter plot of the off-frequency slopes estimated here against the corresponding mean-slope of low-level portions of the on-frequency curves can be seen in Fig 5, with each point corresponding to a single listener. The observed R^2^ of 0.31 was low, but statistically significant (*p < 0*.*05*). The average difference between the linear slopes of the on- and off-frequency TMCs was -0.06 and was not statistically significant (paired-t-test, p = 0.096) with a standard deviation of 0.14.

If the linear portion of on-frequency TMCs can be used to predict the slope of off-frequency TMCs, BM I/O curves may be estimated using only on-frequency TMCs. To examine this, estimates of the lower compression thresholds (i.e., KP values) from the three-section fits to the on-frequency TMCs were compared to the KP values from the BM I/O fits estimated from both on- and off-frequency TMCs. The latter were distributed similarly to data reported in the literature. The average KP in NH listeners was 34 dB SPL (comparable to [2]) and the correlation between the estimated KP and the corresponding hearing-threshold (for all listeners) was high and statistically significant (R^2^ = 0.9, *p < 0*.*001*). Out of 57 BM I/O fits, it was possible to estimate the KP in 20 cases, which is consistent with the fact that not all estimated BM I/O curves show the KP (e.g., [22]).

On average, the KP value fitted to a BM I/O curve could be well approximated by the KP estimate from the corresponding on-frequency TMC. The mean estimate error was 1.0 dB and was not statistically significant (paired t-test, p = 0.69). However, the standard deviation of the on-frequency TMC-based-estimate error was rather high at 9.2 dB and the correlation between the two KP-estimates was moderate (R^2^ = 0.51, *p < 0*.*01*, *n = 15*). On-frequency TMC-based KP estimates could be obtained in 15 cases out of 20 obtained with the standard method. For the CR, the estimates, based on ratio of fitted slopes of the on-frequency TMCs, were not correlated with those from BM I/O curves (R^2^ = 0.02).

Since it is not possible to derive reliable BM I/O parameter estimates from on-frequency TMC alone, some information about the off-frequency TMC is required. If the off-frequency TMC is linear, then only the slope influences the estimated BM I/O curve. Therefore, the second hypothesis tested was that BM I/O parameters estimates can be derived from the on-frequency TMC data and the average slope of the off-frequency TMCs, measured across all listeners, which was 0.26 dB/ms. This hypothesis is similar to that from [23], where a compression-related estimate was based on the on-frequency TMC alone, and relies on two assumptions. First, that off-frequency TMCs can be well approximated with a straight line and second—that the average slope measured across listeners can approximate slopes of individual off-frequency TMCs. While findings of [7] support the first assumption, the same authors noted a large variability of the off-frequency TMC slopes across listeners.

**Fig 6:**
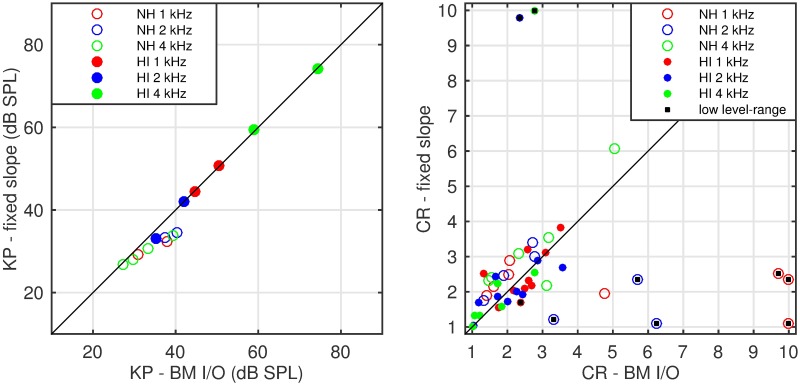
Comparison of the KP and CR estimates from experiment 1. Left panel: a comparison of knee-point estimates. The reference (abscissa) is taken from BM I/O fits involving individual on- and off-frequency TMCs and the ordinate shows knee-points taken from BM I/O fits involving individual on-frequency TMCs and across-listeners slope of the off-frequency TMC. A very good correspondence occurs, with a significant but limited bias (-2.1 dB, *p* = 0.004) and significant correlation (R^2^ = 0.98, *p* < 0.001). Right panel: a comparison of the CR estimates. The open and closed circles show NH and HI listener data, correspondingly. Colours (red, green, blue) code the signal frequency (1, 2 and 4 kHz). Black squares correspond to estimates with a limited dynamic range of the underlying data (see Discussion).

Fig 6 presents the comparison of KP and CR values fitted to BM I/O curves estimated from individual on-frequency data and an average off-frequency TMC slope with the corresponding reference parameter-fits from BM I/O curves based on individual’s on- and off-frequency TMCs. While the KP-fits (left panel) are slightly biased (mean error = -2.1 dB, p = 0.004), the correlation coefficient is very high (R^2^ = 0.98, *p*<0.001, n = 14).

Thus, while KPs may be reliably estimated from on-frequency TMCs and an across-listener average off-frequency TMC slope, CR cannot. However, a closer inspection of the scatterplot depicting CR estimates reveals two groups of points. The first group consists of several points along the diagonal. The second group consists of single data points located far from the diagonal, often at the extreme values of either of the coordinates. If the two groups could be distinguished, one could improve the reliability of the CR estimates.

### Discussion

In the present study, KP levels of BM I/O function were derived using TMCs, in HI and NH listeners. The measurement method used was based on the SIUD paradigm, which is more time efficient than comparable AFC designs [13]. In the case when individual off- and on-frequency TMC data were used to approximate BM I/O, the results were consistent with those from [2] for NH listeners, from [5] for the group of mild-to-moderate HI listeners, and from [6] for NH and HI listeners.

First, it was shown that the slope of the low level part of the on-frequency TMC and the corresponding off-frequency TMC slope are correlated. Therefore, in pursuit of improving the time-efficiency of the TMC experiments, two approaches were tested that avoided measurement of the individual off-frequency TMC. In the first approach, KP estimates were estimated from on-frequency TMC alone. In the second approach, estimates were derived using the across-listener average of the off-frequency TMC slope. BM I/O estimates from complete (on and off-frequency TMC) individual data were taken as the reference. In both cases, the estimate bias was limited (1 and -2.1 dB, for the first and second approach) and significantly correlated (R^2^ = 0.51 and 0.98, respectively) with the reference KP. Both methods returned a KP estimate in, roughly, 3 out of 4 cases, when an estimate was available from the reference fit. The within-listener variation in fits may be attributed to the variability of the off-frequency TMC data not accounted for in the examined approaches, since the curve-fitting strategies were very similar between methods. These results suggest that the straight-line approximation of the off-frequency TMC may be limited. However, the reference method could only return a KP estimate in 20 out of 57 tested cases.

The lack of a correlation between CR estimates obtained from BM I/O functions (derived from both on- and off-frequency TMCs) and from the two alternative approaches is an unexpected result. However, note that some of the CR estimates were close to 10, which was the upper limit of one of the constraints used in the fitting algorithm (see right panel of Fig 6). While Fig 6 only shows comparison between the reference method and the approach of using average off-frequency TMC slopes, similar behaviour was observed using the other approach.

**Fig 7:**
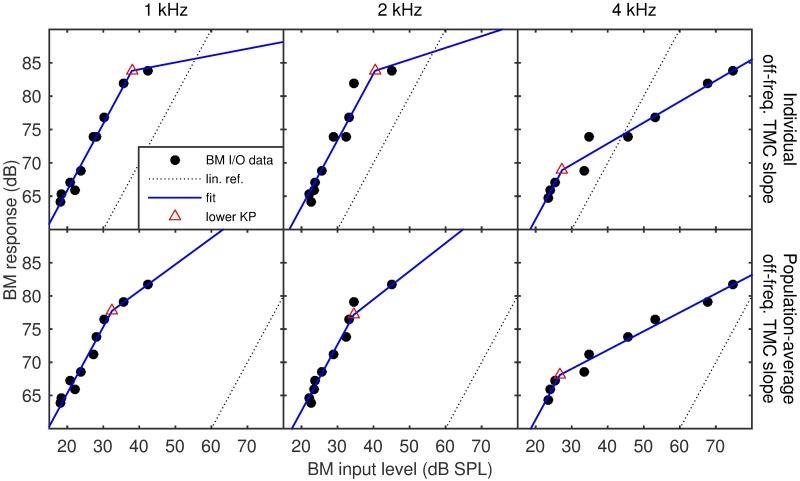
Individual data (listener 5) depicting the variability of the compression ratio estimates. The top panels show the BM I/O data obtained from the reference method, for three tested signal frequencies, together with the obtained fits for one individual listener (listener 5). The lower panels show the corresponding BM I/O data and fits from the average-slope method. The CR estimates for the three tested frequencies are 9.7, 5.7 and 3.2 (reference method) and 2.5, 2.4 and 3.5 (average-slope method), respectively. The latter are closer to what could be expected from the literature for a NH listener (CR 3–5). The maximum on-frequency masker level tested for signal frequencies of 1, 2 and 4 kHz was 42, 45 and 75 dB SPL (respectively), while the input level range above the KP fitted in the reference method was 4, 5 and 47 dB.

To better understand the large mismatch between CR estimates from the reference and the average off-frequency TMC slope method, the distribution of differences between the corresponding CR estimates for all listeners was examined as a function of the maximum masker level that was tested in the on-frequency condition. When the maximum masker level tested was low (less than 50 dB SPL) and/or the range of on-frequency masker levels above the fitted KP was below 15 dB, only a small portion of the observed on-frequency TMC corresponded to the compressive response. Thus, variance in the on- and off-frequency threshold estimates could substantially increase the variance of the CR estimates. In contrast, when the maximum masker-level was high, more data points from the compressive region were available resulting in a more reliable (i.e., lower variance) fit. The described behaviour is best shown in data of listener 5 (Fig 7), where the BM I/O estimates for 1 and 2 kHz include at most two points above the KP. The CR estimates for the three tested frequencies are 9.7, 5.7 and 3.2 (reference method, top 3 panels) and 2.5, 2.4 and 3.5 (average off-frequency TMC slope method, bottom panels), respectively. The latter are closer to what could be expected from the literature for a NH listener (CR 3–5). The maximum on-frequency masker level tested for signal frequencies of 1, 2 and 4 kHz was 42, 45 and 75 dB SPL (respectively), while the input level range above the KP fitted in the reference method was 2.5, 4.7 and 47.4 dB. This is not to suggest that BM I/O fitted with the average off-frequency TMC yields more reliable results than the reference method, but rather that the reliability of both methods suffers when a low range of masker-levels above KP is tested.

Based on the above considerations, some CR estimates were identified as unreliable. The criteria used were whether the maximum masker level tested was less than 50 dB SPL and/or the range of on-frequency masker levels above the fitted KP was less than 15 dB. The cases meeting any of the criteria are marked in the right panel Fig 6 as black squares. A re-analysis of the remaining estimates was conducted. The comparison of the CR estimates from the average off-frequency TMC slope and the reference methods shows an insignificant bias (0.12, paired-sample t-test *p* = 0.28), limited standard deviation of the estimate error (0.7) and a moderate, but significant correlation (R^2^ = 0.5, p < 0.001). The CR, averaged across NH listeners, was 2.5 for the standard method and 2.8 for the average-slope method. The corresponding standard deviations were 1.2 and 1.1. These values are within the lower range of values reported in the literature (CR = 3–5).

In conclusion, the discussed variability of CR estimates is an important practical limitation, particularly since the standard method does not guarantee testing a sufficient dynamic range of masker levels (and hence BM I/O function). The main reason is that thresholds measured for listeners in TMC tasks depend on several factors, e.g., signal level, tested masker-signal interval, listener’s experience with masking tasks, listener’s internal criterion and the shape of the investigated BM I/O function [7]. Consequently, it is not possible *a priori* to define a small set of masker-signal gaps that is guaranteed to span a large dynamic range for many different listeners. Potential solutions based on adaptive strategies (e.g., increasing the gap until certain threshold level is measured and/or using logarithmically distributed masker-signal gaps) lead to unpredictable experimental durations, especially in non-optimal cases.

## Experiment 2: I/O functions estimated with the “gap” method

### Rationale

An important finding from Experiment 1 was that the standard TMC method does not guarantee an effective measurement of the CR due to lack of sufficient control over the tested level range. This makes the paradigm unsuitable for applications where measurement time is at a premium. This limitation of the standard TMC method is a direct consequence of the parameterization of the masker-signal intervals. A further consequence of this is that, during TMC experiments, masker tones are occasionally presented at uncomfortable levels, e.g., as a result of choosing a masker-signal interval that is too large, or when using an adaptive tracking procedure within the experimental paradigm (e.g., transformed up-down). However, if the masker-signal interval was varied (and the masker level was parameterized), the experimenter could ensure that stimuli are never presented at excessive levels. Further, the experimenter could focus on selected sound pressure levels. In this experiment, the consequences of parameterizing the masker level were examined. While a similar approach has been used by [14], they focused on investigating shifts of BM peaks at high levels and did not consider I/O functions of single points on the BM. The procedure of investigating gap thresholds for fixed masker intensities will here be referred to as the “gap method”.

Here, it is assumed that a BM I/O function at a single frequency, and corresponding TMCs, are smooth, bijective, monotonically increasing functions. Moreover, it is assumed that, in the case of sensorineural hearing losses, maximum compression occurs within the 50–75 dB SPL input level range. This is based on the assumptions that the average value of KP in NH listeners is at about 30 dB SPL (e.g., [2]) and, that in some cases, the function returns to linearity above 85–90 dB SPL [2, 5].

### Method

#### Listeners

Individual ears from three NH and three HI listeners were tested. Two of the HI listeners had also participated in Experiment 1 and their data points for the standard method were taken from that experiment. For the on-frequency TMCs collected in Experiment 1, these listeners had been tested at high maximum levels. The remaining three listeners were asked to perform the measurements using both paradigms of Experiments 1 and 2. All listeners provided written informed-consent and the study was approved by the National Research Ethics Committee of Denmark.

#### Stimuli and procedure

The hardware and the parameters used for the standard procedure were the same as in Experiment 1. No cueing masker-signal pair was used. The initial value of the masker-signal interval was a random number taken from a uniform distribution between 95 and 105 ms. The starting step size was set to 6 dB (along the time axis) and was reduced by 1 dB after a down reversal, until a 3 dB step size was reached. Up to 12 responses with the 3 dB step were collected and their geometric mean was taken as a threshold estimate. The minimum allowable masker-signal gap tested was 1 ms. All runs where the adaptive procedure reached the 1 ms border 3 times were terminated and the data from that run were discarded. For each listener, the masker levels were selected before the experimental runs. In the on-frequency conditions, the levels were chosen depending on the signal level used and the hearing impairment status. The maximum masker level was 70 and up to 95 dB SPL for NH and HI listeners, respectively, and the resolution was chosen such that at least 4 masker levels were tested for this listener at each frequency.

Choosing the levels for the off-frequency case was more difficult, since it is difficult to estimate the y-intercept of the corresponding TMC *a priori*. The minimum masker level of 60 dB SPL was chosen as a first guess, the resolution was 5 dB and the maximum tested level was 85 dB SPL. For HI listeners, if the gap-threshold for masker level of 65 dB SPL could not be reliably estimated, the procedure was restarted, the minimum masker level was set to 70 dB SPL and the resolution was 2.5 dB.

#### Training

All listeners were experienced in forward masking tasks. Nevertheless, they were asked to complete three experimental runs at a single masker level and the standard deviation of the estimates was compared to the criterion of less than 5 ms. In the case of higher values, the training was continued until the criterion was met.

#### Data fitting

The data fitting procedure was similar to the one used in Experiment 1, but differed in two ways. First, the fitting error of the curves acquired was measured and minimized along the time axis. If, for a given masker level, more than one out of three experimental runs was aborted (when it reached the 1 ms. masker-signal gap limit) the level was not considered during fitting to avoid a sampling bias. BM I/O functions were obtained from the data by pairing the on- and off-frequency curves, as in Experiment 1. On- and off-frequency thresholds were linearly interpolated to find corresponding thresholds for each masker-signal interval.

### Results

The upper panel of Fig 7 shows mean threshold estimates from the standard method (open symbols) and the gap method (solid symbols). The data presented is from 3 NH listeners and one representative HI listener. Overall, there is a good, but not excellent, correspondence between methods. In some cases (e.g., the HI listener at 1 kHz) the gap-method thresholds are consistently higher than the reference thresholds, or, equivalently masker-levels at threshold are lower for given masker-signal gap. In other cases (the same HI listener, 2 kHz) thresholds obtained from the gap method are higher than the reference thresholds, at least for the larger gaps. In fact, the three highest-level gap-thresholds (42, 43 and 46 ms) follow an almost vertical line.

**Fig 8:**
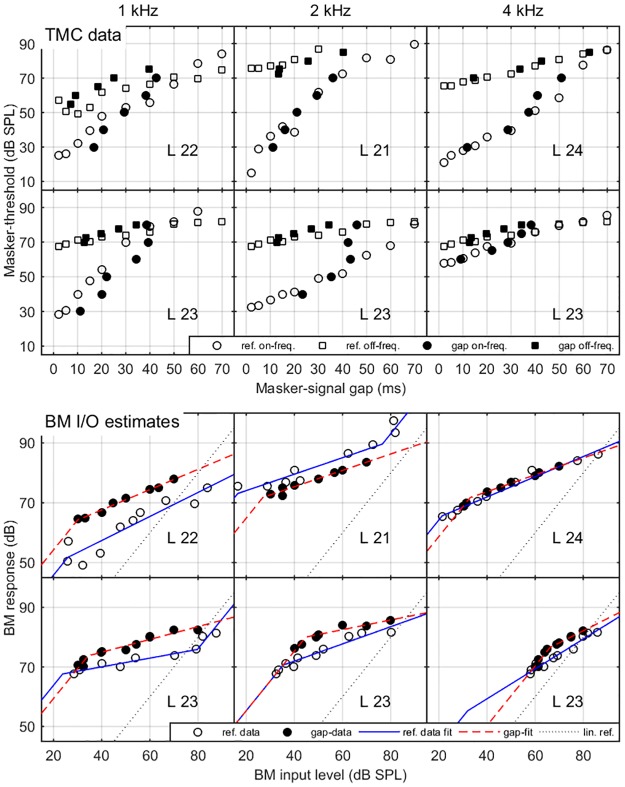
Representative TMC data and corresponding BM I/O estimates from experiment 2. Upper panel: Forward-masking data from the standard method (open symbols) and the gap method (solid symbols). Squares represent the off-frequency data, and circles represent the on-frequency data. Representative NH-listeners’ data is shown in the top three sub-panels, while data from a representative HI listener is shown in the lower sub-panels. Lower panel: BM I/O data estimated from the forward-masking data (open circles—standard method, solid circles—gap method) and the corresponding fits (solid blue and dashed red, respectively). The dotted line shows the linear reference.

The lower part of Fig 8 presents BM I/O estimates from both methods and the corresponding fits. Individual sub-panels correspond between upper and lower panel of Fig 8. Despite the between-method differences visible in the forward-masking data, the BM I/O data shows a clear correspondence between methods (except for a vertical shift visible in all cases except L24). The details of the BM I/O fits are presented next.

**Fig 9:**
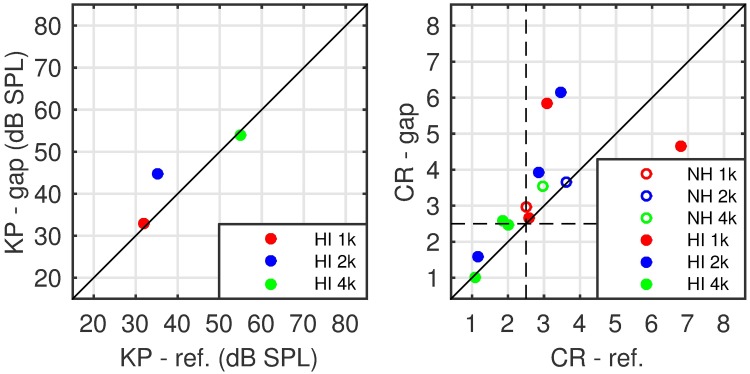
Comparison of estimated basilar membrane I/O characteristics. The left panel presents a comparison of KP estimates obtained with the gap method (ordinate) and the baseline (TMC) method (abscissa). The right panel shows a corresponding comparison of CR estimates. The two dashed lines in the right panel indicate a border that distinguishes cases of near-normal or normal compression (above 2.5, the minimum NH value estimated form the reference method) and abnormal compression. SPL. In both panels NH-listeners’ data is shown with crosses and HI-listeners’ data is shown with circles.

The left panel of Fig 9 shows KP estimates obtained from BM I/O functions derived from the gap-method and from the standard (TMC) method. The right panel shows a comparison of the corresponding CR estimates. The KP estimates obtained with the gap-method are highly correlated to those obtained with the reference method (R^2^ = 0.80), but biased (mean error of 3.1 dB and a standard deviation of 5.6). Since only three KP estimates could be compared between methods, neither correlation nor the bias reached significance. The CR estimates from both methods are significantly correlated (R^2^ = 0.43, p < 0.05). The gap-method CR estimates were biased by 0.59 (not statistically significant using paired-sample t-test, p = 0.14) and the standard deviation of the error was 1.28. As an attempt to classify listeners into two groups (normal vs reduced compression), two straight lines indicate CR equal to 2.5, i.e. the lower limit of compression in NH listeners, estimated from the reference method.

### Discussion

Based on the results of Experiment 1, it is important that the measured TMCs obtained with the SIUD-TMC method span a relatively large level range. In Experiment 2, a modified method, the gap method was tested, where the masker is fixed at different levels and the masker-signal interval (or gap) is varied.

The performance of the gap method was considered in terms of its KP and CR estimates. The KP estimates were highly, but not significantly correlated with the estimates from the reference method, (R^2^ = 0.8), and the KP values estimated with the gap method were biased by 3.1 dB.

As shown in Fig 9, only 3 KP estimates were obtained from both methods. One of the reasons was that the fits obtained for the reference method returned just 4 out of 12 possible KP estimates. The other reason may be related to the way the gap-method samples the portions of the threshold curve in the vicinity of the masker-signal gap and that the minimum allowable gap was 1 ms. If the threshold-gap for a chosen masker level is around 0, the adaptive procedure will not be able to sample the negative gap-values. Thus, it will either overestimate the gap threshold, or the adaptive run will be cancelled. This is analogous to the standard method sampling masker-level thresholds near the maximum allowed masker-level. As a consequence, the gap-method may not be able to sample the lowest-level portions of the on-frequency TMC, which is often a prerequisite for measuring KP level. However, given the high correlation between hearing thresholds and KP estimates reported in the literature and confirmed here, this is not a major concern. On the other hand, varying the masker-signal gap and setting its maximum value to 250 ms assures that the method will converge for the high-level maskers, which, based on results of Experiment 1, is crucial for the estimation of CR.

The gap-based CR estimate was consistently higher than the corresponding estimate obtained with the reference method, with just one exception, where both methods returned an estimate above 4, i.e. in the normal-hearing range. Hence the gap-based estimate was considered biased. Although this bias was moderate at 0.59. the standard deviation of the error estimate was considerable at 1.28. The correlation between the estimates from the two methods was only moderate (R^2^ = 0.43), but statistically significant. Further, it was difficult to reliably classify the CR estimates as normal or impaired based on the gap-method results. Specifically, for the case of the reference method there were 4 cases where the CR estimate for an HI listener was less than the lowest CR estimate for NH listeners (2.5). Out of these 4 cases, the gap method could correctly identify only 3. The remaining case returned an over-estimated CR value, as can be seen in the right panel of Fig 8.

There are several possible sources of the positive bias in the gap-based CR estimate. One may be related to the positive bias in the gap method masker-level threshold estimates, for higher masker-signal gap values, as seen in listener’s 23 data (2 kHz). This positive bias directly leads to overestimation of the CR value. The source of the gap-dependent positive bias in the forward-masking data from the gap method is not clear. The stimuli presented in both methods were identical. Further, a constant shift in the response criterion would have manifested in a horizontal shift of the entire threshold curve.

The bias may be related to the log-spacing of the masker-signal gap in the gap-method, which limits the resolution in the larger masker-signal gap range. The tracking procedure used 3 dB step sizes in adjusting masker-signal gaps. Thus, if one of the gaps tested was 45 ms, the adjacent shorter and longer gaps tested were 32 and 64 ms, respectively. Assuming a psychometric function, with a 50% point around 45–55 ms and 100% performance at 64 ms, the resulting gap-threshold estimate will be underestimated due to the limited resolution. Given that the training criterion was that the maximum standard deviation of 3 consecutive runs was 5 ms, the discussed scenario is not unrealistic. Since gap-thresholds larger than 40 ms were rarely reached in the off-frequency case (see Fig 8), the sampling bias affected the on- and off-curves differently. In summary, the log-spacing could explain both the masker-level dependent bias in the on-frequency TMCs and higher CR estimates obtained from the BM I/O fits.

However, there is a further source of the CR-bias that is inherent to the method that is related to variability of the threshold estimates being distributed along the horizontal axis. If one simulates this variability as Gaussian noise with mean equal to 0 and standard deviation of σ, N(0, σ), then, for a simple case of thresholds acquired for just two masker levels differing by 1 dB, and assuming the “true” slope of the threshold curve to be S, the corresponding slope estimate has a distribution of a reciprocal of N(1/S, 2σ). Such a distribution does not have finite integer moments. In particular, the expected value is infinite [24]. Moreover, if S increases, 1/S tends to 0 and the estimate of S tends to infinity. Thus, this contributes to the positive bias and high variability of the CR estimates.

## General discussion

The purpose of the two experiments was to investigate possible means of improving measurement efficiency of TMC paradigms. In Experiment 1, there were two key findings. First, the lower slope of the on-frequency TMC was statistically the same as the overall slope of the off-frequency curve. This suggested that it was possible to disregard the measurement of the off-frequency curve to obtain an approximation of BM I/O parameters such as KP and CR. BM I/O curves estimated using only on-frequency TMC data were compared to BM I/O curves estimated using both on- and off-frequency TMC data. While the KP estimates from both methods were consistent on average (non significant bias of 1 dB) the standard deviation of the error was rather large (9.2 dB) and correlation was moderate, with R^2^ = 0.51. Moreover, the CR estimates from both fits were not correlated.

Therefore, it was concluded that some information about the off-frequency TMC must be included in the fit. Off-frequency TMCs can often be well approximated by a straight line (e.g., [7]), which is described by two parameters—the slope and the intercept. Since the intercept only affects the vertical positioning of the TMC-derived BM I/O, the slope is more important for the KP and CR estimates. Based on the data from 20 listeners tested in Experiment 1, the average slope of an off-frequency TMC was found to be 0.26 dB/ms. Three-section functions were fitted to BM I/Os estimated from individual on-frequency TMC data and off-frequency TMC thresholds based on the population-average slope. The resulting KP and CR estimates were compared to the reference values obtained from BM I/O fits based on individual on- and off-frequency thresholds. While the average-slope-based KP-fits (left panel) were slightly biased (mean error = -2.1 dB, p < 0.05 and highly correlated R^2^ = 0.98), the correlation of CR estimates from both methods was again low R^2^ < 0.01, which is a critical issue. While reliable KP estimates can be obtained from audiogram data alone, CR estimates cannot.

Closer inspection of the CR-scatterplot and analysis of the corresponding TMC data revealed a source of the low correlation between the two estimates. It was found that the reliability of CR estimates was low if the maximum on-frequency masker-level was lower than 50 dB or the dynamic range of the masker-levels measured above the fitted KP was lower than 15 dB. Thus, CR estimates are not reliable unless a sufficiently large portion of the compressive region is tested. Re-analysis of the results that included only cases where a sufficient dynamic range was tested revealed that the CR estimates from the average-slope and the reference methods showed an insignificant bias (0.12, paired-sample t-test *p* = 0.28), limited standard deviation of the estimate error (0.7) and a moderate, but significant correlation (R^2^ = 0.5, p < 0.001). The CR, averaged across NH listeners, was 2.5 +/- 1.2 for the reference method and 2.8 +/- 1.1 for the average-slope method. These values were within the lower range of values reported in the literature (CR = 3–5). A plausible reason for the low estimates was that the masker frequency in the off-frequency condition was 2.4 kHz, with signal frequency equal to 4 kHz and such off-frequency curve may be itself subject to compression factor of up to 2:1 [20].

The findings of Experiment 1 led to the conclusion that it is not possible to reliably estimate the KP and CR values without testing a sufficiently large range of on-frequency masker levels. Given high variations between individual listeners, such as response criterion, detection efficiency, it is not possible to pre-select a set of masker-signal gaps that would ensure testing of a sufficient dynamic range. Therefore, in Experiment 2, an alternative paradigm, the gap method, was introduced and compared against the standard TMC. The gap-method was designed to enable testing masker-signal gap thresholds for high masker levels.

Within the gap method and the reference method, the estimates of KP and CR were derived from the BM I/O curves. It was found that KP estimates obtained from the gap-method were highly correlated with those based on the standard TMC paradigm. However, the correlation was not significant due to very low number of compared estimates. This is likely due to the fact that the gap method is not well suited for measuring the lower portion of the on-frequency TMC curve. However, due to predictability of KP based on hearing-thresholds, this is not seen as a major limitation.

The estimates of BM compression, based on the TMC and gap methods were correlated (R^2^ = 0.43, p < 0.05). The gap-method CR estimates were biased by 0.59 (not significant, paired-sample t-test p = 0.14) and the standard deviation of the error was 1.28. While the observed bias was not statistically significant, two important factors may lead to overestimation of CR when using the gap method. First, using logarithmic masker-signal gap spacing may lead to underestimation of gap-threshold for large gaps (around 40–60 ms). This results in overestimation of on-frequency TMC slope. Since the off-frequency gap-thresholds rarely reached values above 40 ms, the sampling-bias acted differently on the on- and off-frequency curves, leading to overestimated CRs. The second possible source of bias in CR estimates is inherent to the method. Due to varying the masker-signal gap the distribution of slope estimates may become similar to the reciprocal of a Gaussian distribution, which has an infinite mean.

The relative contribution of these two factors is unclear. Given that the overall bias was limited and not statistically significant suggests the second factor did not play a major role. If it did, a larger magnitude of bias could be expected. We speculate that measuring gap-thresholds for more than two closely spaced masker-levels, could effectively limit the resulting estimate bias. Additional experiments, employing uniformly-sampled masker signal gaps could clarify the picture.

Regarding time-efficiency, the gap-method ensured testing the large masker-levels for each listener and condition while using at most 7 unique masker-levels. This is an improvement over the standard method used in Experiment 1, where testing 10 masker-signal gap values wasn’t always sufficient for sampling the high-level portions of the on-frequency TMC. On the other hand, the gap-method could not return reliable threshold estimates for low-level portions of the TMC, due to a sampling bias when measuring very small gaps (i.e., near 0 ms). This is of little consequence for NH listeners or HI listeners with near-normal hearing-thresholds, given the higher importance of measuring CR than KP. However, in the case of HI listeners with higher hearing-thresholds, the sampling bias may lead to an inability of testing portions of the on-frequency TMC where residual compression is still present. This, in turn, may lead to erroneous estimate of listener’s BM I/O.

In summary, each of the tested methods has different advantages and disadvantages. The standard method is better at measuring the lower level portion of the on-frequency TMC. However, the gap-method offers superior control over the tested dynamic range. This allows for more predictable experimental durations and assures measuring compressive regions of the BM I/O if they are present.

## Summary and conclusion

While it is possible to estimate individual BM I/O functions using forward masking paradigms such as TMC, they are very time consuming. The aim of the present study was to investigate two approaches that could, potentially, improve the time- and measurement-efficiency of the TMC experiments, making them feasible for use as a tool to better characterizing individuals’ hearing loss in other research experiments. This would allow researchers to increase the homogeneity of test groups and/or allow more fine-grained investigations into the perceptual consequences of hearing loss from different etiologies. In Experiment 1, it was investigated whether it is possible to derive BM I/O estimates from on-frequency TMCs, without measuring the linear reference off-frequency TMC. The first finding was that the slope of the low-level portion of the on-frequency TMC can, on average, approximate the slope of the off-frequency TMC. However, the variability of individual CR estimates suggests this is likely not practical. The second finding was that, provided a sufficient range of on-frequency masker levels are tested, reliable estimates of CR can be obtained using an across-listen average off-frequency TMC slope. In Experiment 2, TMCs were measured by finding gap thresholds as functions of parameterized masker levels, which is an inversion of the standard TMC paradigm. The gap-method’s design allows for better control over the dynamic range and the measured bias of KP and CR estimates was not significant. As the most likely source of the CR bias was the log-spacing of the tested masker-signal gaps, it is likely that uniform spacing could remove a large portion of the bias. One can speculate that since the standard TMC and the gap-method show relative advantages in the low-gap and high-masker-level portions of the TMC, respectively, a combination of both (with linear sampling in gap and level dimensions) could be useful.
